# A systematic review on robot-assisted language learning for adults

**DOI:** 10.3389/fpsyg.2024.1471370

**Published:** 2024-11-06

**Authors:** Qi Deng, Changzeng Fu, Midori Ban, Takamasa Iio

**Affiliations:** ^1^School of Foreign Studies, Northeastern University, Qinhuangdao, China; ^2^Sydney Smart Technology College (SSTC), Northeastern University, Qinhuangdao, China; ^3^Hebei Key Laboratory of Marine Perception Network and Data Processing, Northeastern University at Qinhuangdao, Qinhuangdao, China; ^4^Intelligent Robotics Laboratory (IRL), Osaka University, Osaka, Japan; ^5^Faculty of Culture and Information Science, Doshisha University, Kyoto, Japan

**Keywords:** robot-assisted language learning, adult education, AI in education, language learning effectiveness, human-robot interaction

## Abstract

In the 21st-century era of globalization, language proficiency is a pivotal connector across cultures, with artificial intelligence (AI) revolutionizing educational paradigms through Robot-Assisted Language Learning (RALL). This systematic review examines the role of RALL in adult second language acquisition, focusing on its pedagogical strategies and learner engagement. Unlike the previous systematic reviews that explore the multifaceted roles of robots in language learning, including as teachers, tutors, assistants, and peer learners, we identify explicit and implicit instructional strategies within RALL, highlighting the unique learning landscape of adult learners characterized by self-regulation and self-direction. We assess the latest advancements in RALL for adult learners through three research questions, compare the effectiveness of explicit versus implicit instructions, and investigate affective factors enhancing RALL performance. Our review contributes a comprehensive status analysis, in-depth exploration of interaction modes, and insights for future research directions, providing a roadmap for academic research and practical guidance for educators and robot developers. This study aims to optimize RALL strategies to better meet the needs of adult learners, fostering a more efficient and engaging language learning experience.

## 1 Introduction

In the era of 21st-century globalization, proficiency in languages has emerged as an essential connector across the globe; it serves not only as a catalyst for personal career advancement but also as a conduit for cross-cultural dialogue. The incorporation of cutting-edge artificial intelligence into education has given rise to an innovative pedagogical tool, Robot-Assisted Language Learning (RALL). RALL harnesses advanced algorithms and human-computer interaction technologies, empowering robots to act as efficient educators or engaging conversationalists, thereby supporting learners in their acquisition of linguistic expression and comprehension. The advent of this novel approach represents a pivotal transformation in language learning methods, bringing new ideas into conventional educational frameworks.

### 1.1 Scope and objectives

Our research narrows its focus to adult second language acquisition within the RALL framework, emphasizing the comparative effectiveness of explicit and implicit instructional strategies. This targeted scope is essential because adult learners have distinct learning requirements compared to children. Unlike children, adults bring a complex set of dynamics to the learning process, often seeking more self-directed and contextually rich educational experiences that traditional instruction methods may fail to provide. This reviews is poised to scrutinize the manner in which RALL can be leveraged to accommodate the sophisticated styles of adult learners. The objective is to elucidate and enhance instructional strategies for RALL that foster autonomy, critical thinking, and the tangible application of linguistic competencies–elements that are indispensable for adults in the language acquisition process. By doing so, we aspire to augment the efficacy of language education within this demographic, thereby contributing to a more impactful and resonant pedagogical approach for adult learners in the RALL environment.

### 1.2 Assessing the current instructional strategies in RALL and their limitations

Previous systematic reviews (Randall, 2019; Belpaeme et al., 2018; Van den Berghe et al., 2019; Engwall and Lopes, 2022) have qualitatively summarized the burgeoning field of RALL, highlighting the multifaceted roles of social robots and their influence on learners' emotional domains, which can be summarized into the following categories:

- **Robot as a tutor for a student:** in the role of a tutor/assistant, robots offer one-on-one instruction tailored to the individual needs of the student. Robots can adapt to the learner's pace and provide customized feedback, making the learning process more efficient and targeted (Han et al., 2008; Lee et al., 2011; Kennedy et al., 2016; Schodde et al., 2017).
- **Robot as an assistant for a teacher:** the robot serves as a support system for human teachers, assisting with introducing exercises, telling stories, asking the learners questions or answering theirs (Kanda et al., 2004; Park et al., 2011; Alemi et al., 2014, 2015).
- **Robot as a peer learner for a student:** robots act as simulated peers, learning the language together with the human learners; engaging students in conversation and collaborative learning activities (Mubin et al., 2013; Gordon et al., 2016; Mazzoni and Benvenuti, 2015).
- **Robot as a novice being taught by a student:** this innovative role flips the traditional dynamic by having the robot learn from the student, which can enhance the student's understanding and retention of language concepts as they teach and correct the robot, thus reinforcing their own knowledge (Tanaka and Matsuzoe, 2012).

The roles of robots in Robot-Assisted Language Learning (RALL) are intentionally crafted to be inclusive, catering to a spectrum of learners that encompasses both children and adults (Aidinlou et al., 2014; Randall, 2019; Van den Berghe et al., 2019; Belpaeme et al., 2018). However, it is crucial to acknowledge the fundamental differences in how these two groups interact with the language learning process. Children are typically characterized by a higher degree of compliance with instructional directives, a trait that sets them apart from adults, who contribute a more intricate set of dynamics to the learning environment. While instructional methods such as Total Physical Response (TPR) and the Direct Method are effective for children, leveraging their natural mimicry and engagement in interactive activities, these methods reveal limitations when applied to adults. Adult learners often require educational approaches that emphasize self-direction, critical thinking, and the application of language skills in real-world contexts, reflecting their distinct cognitive styles and preferences. The structured and sometimes repetitive nature of children's RALL strategies may not sufficiently address the diverse learning needs and the more complex linguistic requirements of adults. Therefore, the instructional strategies within RALL must be critically examined and adapted to ensure they are responsive to the unique characteristics of adult learners. This includes the development of pedagogical approaches that provide flexibility and cater to varied learning paces, offering opportunities for adults to apply language skills in meaningful and contextually relevant ways (Randall, 2019; Engwall and Lopes, 2022; Lee and Lee, 2022).

### 1.3 Adult learners' unique learning landscape

Adult learners approach language learning with a distinct constellation of motivations, objectives, and levels of cognitive development (Jarvis, 2011; Zhang, 2022; Dornyei, 2013). Moreover, adult learners exhibit lower compliance with direct instructions due to mutual self-regulation (Ho and Lim, 2020; Bakhtiar and Hadwin, 2022). This unique set of factors shapes not only their engagement with the learning material but also significantly influences the outcomes they achieve. The complex nature of adult learning indicates a propensity for approaches that foster autonomy and self-direction, needs that may not be optimally met by the current strategies employed in RALL. The need, therefore, arises for a research focus that shifts toward understanding and optimizing the instructional strategies used by RALL robots, especially in the context of adult language learning.

### 1.4 Research focus on instructional strategies in adult RALL

The existing body of research has shown a significant inclination toward examining the roles robots fulfill within educational settings, often sidelining the type of instructional strategies they should implement. This oversight is particularly evident in the context of adult second language learning, where the efficacy of explicit versus implicit instructional methods has not yet received the scrutiny it deserves.

As shown in Table 1, we defined two main forms of RALL for adults: explicit instruction and implicit instruction.

- **Explicit instruction:** characterized by the direct teaching of language rules and structures, involving top-down teaching methods where the instructor presents knowledge. For example, a robot might directly instruct an adult learner on how to order coffee at a cafe by saying, “To order a coffee, you say, ‘I would like a large cappuccino, please'.” This strategy might not fully resonate with adult learners' preferences. Adult learners may lean more toward approaches that facilitate self-discovery and the internalization of linguistic patterns (Dornyei, 2013).
- **Implicit instruction:** promotes reflection on language learning strategies and performance without overt guidance on language rules, involving bottom-up approaches that encourage learners to discover language patterns through exposure and interaction. For instance, instead of direct instruction, a robot might engage in a role-play scenario at a simulated cafe with other robots, where the learner observes and participates in ordering a coffee without being explicitly told the grammatical structure. This approach allows learners to pick up the language through context and usage, which can be more conducive to developing intuitive knowledge necessary for second language learning (Khalifa et al., 2018).

**Table 1:** Summary of studies on robot-assisted language learning.

**Study**	**Participants**	**Target language**	**Method**	**Robot and role**	**Instruction type**	**Party**
**Vocabulary/ comprehension**						
Kanero et al. (2018)	*N* = 24, age = 20.18	English	1 condition; duration: 20 min	NAO; tutor	Explicit	One-on-one
de Haas and Conijn (2020)	*N* = 60, age = 24.00	Vimmi words	3 conditions: positive/ negative/ no feedback; duration: 1 trial	Pepper; teacher	Explicit	One-on-one
Banaeian and Gilanlioglu (2021)	*N* = 36, age = 20.03	English	2 conditions: RALL/ non-RALL; duration: 1 trial	NAO; teaching assistant	Explicit	In groups
Alimardani et al. (2022)	*N* = 25, age = 22.20	Vimmi words	2 conditions: feedback with/ without gesture; duration: 55-60 min	NAO; tutor	Explicit	One-on-one
Prinsen et al. (2022)	*N* = 8, age = 22.6	ROLIA	2 conditions: adaptive/ random tutoring; duration: 1 trial	NAO; tutor	Explicit	One-on-one
Riedmann et al. (2024)	*N* = 126, age = 20.4	Spanish	4 conditions: 2 non-robot, 2 robot; duration: 14 tasks	Pepper; tutor	Explicit	One-on-one
Vrins et al. (2022)	*N* = 27, age = 21	ROLIA	2 conditions: embodied/ screen; duration: 1 trial	NAO; tutor	Explicit	One-on-one
Gkinos et al. (2022)	*N* = 30; age = 20–60	Pontic dialect	1 condition: RALL; duration: 1 session	NAO; tutor	Explicit	One-on-one
**Pronunciation**						
Krisdityawan et al. (2022)	*N* = 20, age = 21–24	English	2 conditions: RALL/ non-RALL; duration: 3 weeks	NAO; tutor	Explicit	One-on-one
Amioka et al. (2023)	*N* = 27, age = 26	Japanese	3 conditions: matched/ mismatched or default model; duration: 1 trial	Furhat; teacher	Explicit	One-on-one
Zinina et al. (2023)	*N* = 20, age = 24.5	Chinese	2 conditions: gesture cues/ only speech; duration: 1 trial	F-2; teacher	Explicit	One-on-one
**Speaking and communication skills**						
Iio et al. (2019)	*N* = 9, age = 19.3	English	1 condition; duration: 7 days	CommU; tutor	implicit	One-on-one
Iio et al. (2024)	*N* = 26, age = 18–22	English	2 condition; duration: 7 days	CommU; tutor	implicit	One-on-one
Lopes et al. (2017)	*N* = 22; age = 29.1	Swedish	2 conditions: L1 speaker/ moderator then robot/ vice versa; duration: 30 min	Furhat; moderator	Implicit	One-on-one
Engwall et al. (2021)	*N* = 33; age = 32A	Swedish	4 conditions: interviewer/ narrator/ facilitator/ interlocutor; duration: 2-4 trials	Furhat; moderator	Implicit	One-on-two
Engwall and Lopes (2022)	*N* = 33; age = 32	Swedish	4 conditions: interviewer/ narrator/ facilitator/ interlocutor; duration: 2-4 trials	Furhat; moderator	Implicit	One-on-two
Shen et al. (2019)	*N* = 10; university students	English	1 condition; duration: 6 weeks	NAO; teaching assistant	Explicit	In groups
Nomoto et al. (2022)	*N* = 10; age = 20–35	Mandarin Chinese	2 conditions: physical/ virtual agent; duration: 15-20 min	Qilin	Explicit	One-on-one
**Grammar**						
Khalifa et al. (2016)	*N* = 51; age = 18–24	English	2 conditions: with/ without PC monitor; duration: 1 session	NAO; teacher and peer	Implicit	Two-on-one
Khalifa et al. (2017)	*N* = 37; age = 18–24	English	2 conditions: teacher asks first/ robot asks first; duration: 1 session	NAO; teacher and peer	Implicit	Two-on-one
Kanero et al. (2018)	*N* = 16; age = 18–24	English	2 conditions: human learner first/ robot learner first; duration: 1 session	NAO; teacher and peer	Implicit	Two-on-one
Khalifa et al. (2019)	*N* = 80; age = 18–24	English	2 conditions: teacher to robot then learners or learners only; duration: 1 session	NAO; teacher and peer	Implicit	Two-on-one

Therefore, to explore the current application of RALL in adult education, and analyze the potential effect of explicit and implicit instruction, so as to propose future research directions, this systematic review will revolve around the following three research questions, aiming to provide in-depth insights into the application of RALL in adult language learning:

- **RQ1: What are the latest advancements in RALL for adult second language learners**
  This research question aims to assess the academic advancements of RALL in adult education, including its effectiveness and challenges faced, providing foundational data and insights for future research and practice.
- **RQ2: Which is more effective of learning outcomes, in RALL, explicit or implicit instruction?**
  By comparing the effectiveness of the two types of robot interaction in adult language learning, this research question will help us understand which type of robot assistance may be more suitable for different learners, thus guiding the design of RALL systems that better meet the needs of learners.
- **RQ3: What affective factors of robot can enhance the performance of RALL?**
  This research question will explore how the design and interaction characteristics of robots affect learning outcomes, providing a scientific basis for optimizing robot design and improving the performance of RALL.

Based on the aforementioned RQs, this systematic review offers three principal contributions to the application of Robot-Assisted Language Learning (RALL) in adult education through a thorough analysis and discussion of existing literature:

- **Comprehensive status analysis:** this review provides a complete overview of the application of RALL in adult language learning, revealing the strengths and limitations of current RALL practices. By analyzing the outcomes of various studies, this paper offers an in-depth understanding of the current state of RALL, including its impact on learners' speaking skills, vocabulary, pronunciation, and grammar learning. Moreover, this review also explores the roles of different types of robots (i.e., explicit and implicit instructions) in language learning and how they can be adapted to the unique needs of adult learners.
- **In-depth exploration of interaction modes:** the paper delves into the effectiveness of explicit and implicit instruction modes within RALL and how they affect learner engagement and learning outcomes. By comparing teaching strategies and robot roles proposed in different studies, this paper uncovers the significance of implicit learning in adult language learning and offers insights into how to integrate explicit and implicit instruction modes in future RALL system designs.
- **Insights for future research direction:** based on a systematic review of the existing literature, this review proposes specific directions for future RALL research. These research directions include the integration of explicit and implicit instructions, strategies to stimulate learners' intrinsic motivation, methods to promote emotional engagement, mechanisms to enhance social interaction, and approaches to improve cross-cultural adaptability. These recommendations not only provide a roadmap for academic research but also offer guidance for educational practitioners and robot developers.

The remainder of this paper is structured as follows: we introduce the strategy and procedure of our survey work in Section 2; the comprehensive review of related studies is conducted in Section 3; the detailed discussion of RQs and future works is given in Section 4; in Section 5, we give the conclusion of this systematic review.

## 2 Method

### 2.1 Search strategy

In our endeavor to compile a comprehensive review of Robot-Assisted Language Learning (RALL), we embarked on a systematic search for empirical studies. Our search strategy was designed to be both exhaustive and precise, utilizing the expansive database of Google Scholar. We employed the keyword “robot-assisted language learning (RALL)” to filter and retrieve relevant literature up to the year 2024.

**Figure 1:**
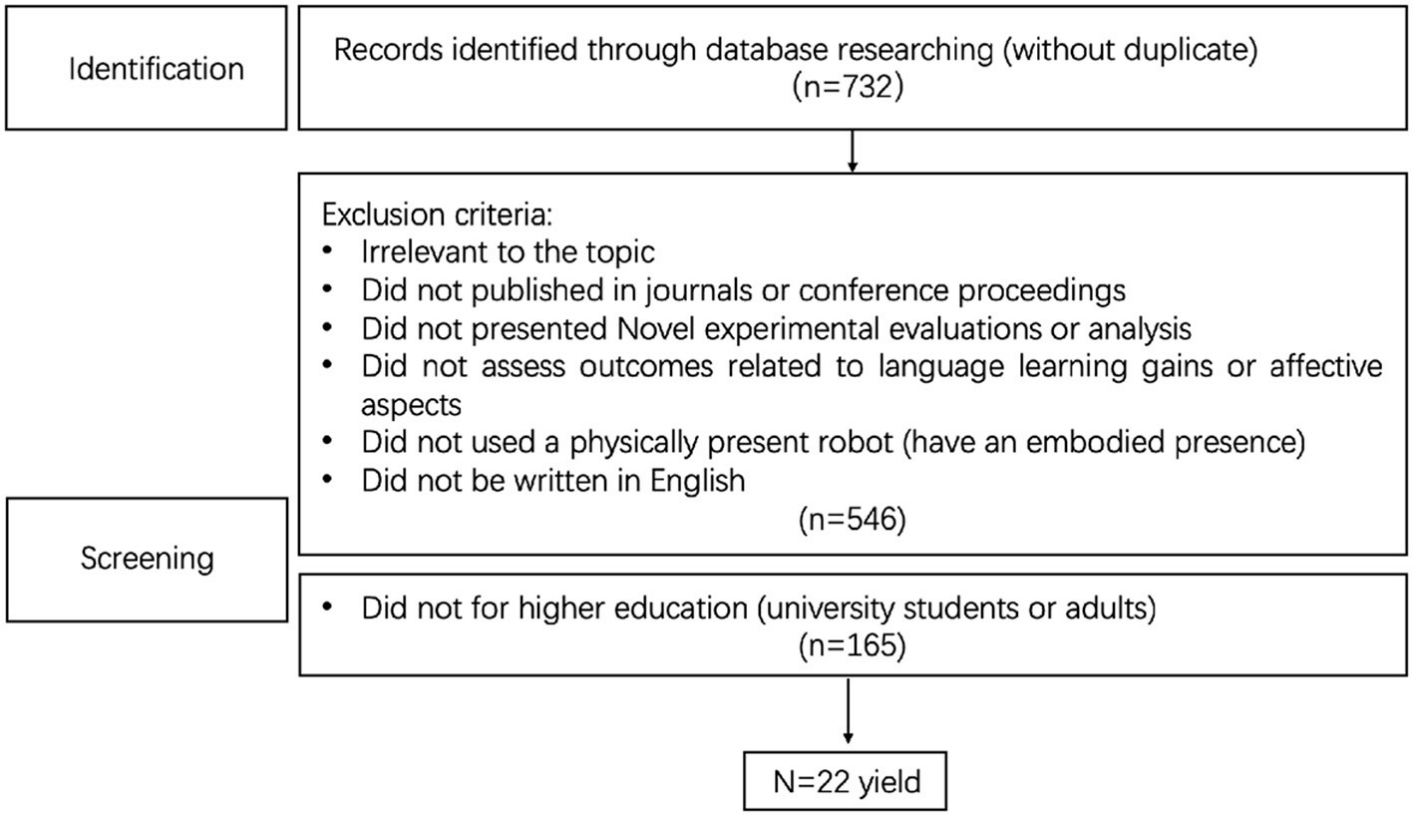
The references selection process of this review.

Figure 1 shows the selection process. The initial search yielded a substantial pool of 732 studies, which represented a wide array of research conducted in the field of RALL. This figure underscored the growing interest and development in the intersection of robotics and language learning. The next step involved a meticulous process of deduplication to ensure that each study considered was unique and had not been inadvertently included multiple times.

### 2.2 Selection strategy

With a clear and focused approach, we developed a set of exclusion criteria to refine our selection process. This strategy aimed to ensure that the studies included in our review were not only relevant but also met specific methodological standards. The criteria were as follows:

1. **Relevance to the topic:** studies that did not directly address the topic of RALL were excluded.
2. **Publication venue:** we focused on studies published in peer-reviewed journals or conference proceedings to ensure academic rigor and credibility.
3. **Novelty:** we excluded studies that did not present novel experimental evaluations or analysis, aiming to include only those that contributed new insights to the field.
4. **Assessment of outcomes:** we were particularly interested in studies that assessed outcomes related to language learning gains or affective aspects, as these are critical measures of RALL effectiveness.
5. **Embodied presence:** studies involving robots with a physically present, embodied presence were prioritized, as they are central to the concept of RALL.
6. **Language:** we limited our search to studies written in English to maintain consistency and accessibility for our review. Target Audience: We further narrowed down our selection to studies related to higher education, focusing on university students or adults, as this demographic represents a significant and relevant user group for RALL. After applying these criteria, 545 records were excluded, significantly narrowing down our pool of studies. An additional 164 records were excluded based on the criterion related to the target audience. This stringent selection process ultimately led to the inclusion of 22 studies that met all the established criteria, forming the core of our review on RALL.

## 3 Literature review

### 3.1 Categorization based on explicit and simplicity instruction

Explicit instruction is characterized by the direct teaching of language rules and structures. This method is often associated with a more structured and controlled learning environment where the teacher or the robot provides clear guidance on what to learn and how to learn it. Kanero et al. (2018) describe a method where the robot first explains the structure of the lesson and then introduces words one by one. The robot defines the target word, asks participants to utter it multiple times, and repeats the definition for reinforcement. de Haas and Conijn (2020) initiate interaction by teaching different words. The robot translates an animal into Vimmi and asks participants to repeat the word, followed by a feedback phase where the participant searches for the target word. Banaeian and Gilanlioglu (2021) provide definitions and examples of words and conduct comprehension checks, ensuring that learners understand the material being taught. Alimardani et al. (2022) involve the robot asking participants to select an image representing the target word and providing feedback on the accuracy of their selection. Prinsen et al. (2022) offer an introduction to the language and experiment setup, followed by the robot repeating word pairs in the target language, with participants instructed to memorize these words and their meanings. Riedmann et al. (2024) structure the learning environment into theoretical blocks with exercises, providing direct, constructive feedback to learners. Vrins et al. (2022) vocalize words, state their meanings, and perform gestures for repetition, enhancing the learning experience. Gkinos et al. (2022) provide recorded instructions and feedback based on the given answers, guiding learners through the learning process. Krisdityawan et al. (2022) give learners instructions for each lesson, including gestures, and ask them to imitate the intonation and rhythm in prosody lessons. Amioka et al. (2023) translate words into Dutch, followed by repetitions in Japanese, with participants asked to repeat the Japanese word. Zinina et al. (2023) provide direct learning instructions and feedback, ensuring clarity in the learning objectives. Shen et al. (2019) assist in classroom spoken English practice, including word repetition and text reading, reinforcing language acquisition through practice. Nomoto et al. (2022) give emotional responses based on user performances.

Implicit instruction, on the other hand, promotes reflection on language learning strategies and performance without overt guidance on language rules. This approach is more conducive to developing intuitive knowledge and encourages learners to discover language patterns organically. Lopes et al. (2017), Engwall et al. (2021), Engwall and Lopes (2022) describe a language café type of interaction where the robot engages in social conversations with participants on any topic of their choice, fostering a natural language learning environment. Khalifa et al. (2016, 2017, 2018), Kanero et al. (2018), Khalifa et al. (2019) involve a teacher robot asking a question to a learner robot, with the user listening to the conversation. This method allows learners to pick up language through observation and listening. Iio et al. (2019, 2024) play the role of the user's dialogue partner without providing any learning suggestions.

The categorization of these studies into explicit and implicit instruction sheds light on the diverse approaches to language learning in RALL. Understanding these methods can help educators and researchers tailor their teaching strategies to better suit the needs and preferences of adult learners, ultimately enhancing the effectiveness of language acquisition.

### 3.2 Learning gains in robot-assisted language learning

#### 3.2.1 Vocabulary

In the realm of vocabulary acquisition research, studies have not only investigated how learners' attitudes toward robots, personality traits, and cognitive states influence learning effectiveness but also explored how robots can facilitate learning through interaction and feedback (Tables 1, 2).

**Table 2:** Evidence of categorization to explicit or implicit instruction.

**Study**	**Evidence for categorization**
**Explicit**	
Kanero et al. (2018)	Direct teaching with word definitions, repetition, and definitions checks.
de Haas and Conijn (2020)	Word teaching through repetition and feedback on word searches.
Banaeian and Gilanlioglu (2021)	Definitions and examples provided with comprehension checks.
Alimardani et al. (2022)	Image selection and direct feedback on word association.
Prinsen et al. (2022)	Introduction and repetition of word pairs with memorization instructions.
Riedmann et al. (2024)	Structured learning blocks with direct feedback on exercises.
Vrins et al. (2022)	Word vocalization, meaning explanation, and iconic gestures for repetition.
Gkinos et al. (2022)	Recorded instructions and feedback on answers.
Krisdityawan et al. (2022)	Gesture-assisted lesson instructions and intonation imitation.
Amioka et al. (2023)	Translation and repetition of words with direct instruction.
Zinina et al. (2023)	Direct learning instructions and feedback.
Shen et al. (2019)	Classroom assistance in spoken English practice.
Nomoto et al. (2022)	Emotional response to user performances.
**Implicit**	
Lopes et al. (2017), Engwall et al. (2021), Engwall and Lopes (2022)	Social conversation without explicit language instruction.
Khalifa et al. (2016, 2017, 2018, 2019)	Conversational model where learner listens to teacher-learner interaction.
Iio et al. (2019, 2024)	Dialogue partnership without learning suggestions.

Firstly, concerning the influence of learners' attitudes, personality traits, and cognitive states on learning outcomes, Kanero et al. (2018) conducted a study on the impact of individual attitudes and personality traits on adult second language (L2) vocabulary learning, with a particular focus on robot-assisted instruction. The research involved 24 Turkish-speaking adult students who completed English proficiency tests, personality difference questionnaires, and immediate and delayed vocabulary tests both before and after receiving one-on-one English instruction from the NAO robot. The findings indicated that a negative attitude toward robots, especially concerns regarding the potential negative societal impact of robots, was negatively correlated with vocabulary learning outcomes (*p* < 0.05), while only the personality trait of openness was significantly associated with delayed receptive test scores (*p* < 0.05). Further analysis using a regression model confirmed that the negative attitude toward the societal impact of robots was the sole significant predictor of learning outcomes. These results imply that an individual's attitude toward robots might exert a more substantial influence on their success in robot-assisted language learning compared to general personality traits, offering valuable insights for the development of future personalized language learning tools. Banaeian and Gilanlioglu (2021) investigated the impact of the NAO robot as a teaching assistant on students' vocabulary learning and their attitudes in university English classes. The study found that while there was no significant difference in vocabulary test scores between the experimental group (which used the NAO robot) and the control group (which did not use the robot) (*p* = 0.391), the majority of students expressed positive attitudes toward the NAO robot, considering it to be an intelligent, safe, and helpful learning tool. The NAO robot enhanced students' understanding and memory of new vocabulary through interaction, providing contextual examples, repetitive practice, and immediate feedback, thereby promoting vocabulary learning to some extent. However, there were also some challenges, such as issues with voice recognition, excessively fast speech rates, and distraction due to the novelty of the technology. The study suggests that before integrating Robot-Assisted Language Learning (RALL) technology into the classroom, its advantages and limitations should be thoroughly considered, and professional training should be provided to teachers to more effectively utilize this tool to assist in vocabulary teaching. Riedmann et al. (2024) studied the application of social robots and gamification in adult vocabulary learning of Spanish and their effects on learning motivation, engagement, and performance. The researchers developed a technology-enhanced learning environment that integrates the social robot Pepper and gamification elements to explore their individual and combined effects on adult learners. However, the study found that the introduction of social robots did not significantly improve learners' vocabulary learning outcomes (*F* = 0.018, *p* = 0.668) and even led to lower engagement (*F* = 6.48, *p* = 0.012, η^2^ = 0.05). The results indicate that the integration of social robots and gamification elements in adult language learning environments requires more in-depth and meticulous design, and their enhancement of learning outcomes is not immediate. Although these technologies offer new learning experiences, they did not show the expected effect in improving vocabulary learning, suggesting that future research in this field needs to focus more on how to effectively integrate these tools to enhance language learning efficiency.

When examining learners' cognitive states, in addition to subjective methods such as self-reported questionnaires, recent research has also emerged that utilizes objective data from brain-computer interfaces (BCI) to detect electroencephalogram (EEG) signals to verify the effectiveness of Robot-Assisted Language Learning (RALL) for adults. Prinsen et al. (2022) developed a system that calculates an EEG engagement index in real time by measuring electroencephalogram (EEG) signals from the frontal lobe, serving as a neurophysiological indicator of user engagement. Participants in the experiment learned vocabulary in the ROILA language through interactions with a NAO robot. The study found that under adaptive tutoring conditions, the robot provided additional instructional cues based on changes in the EEG engagement index, with the aim of enhancing learning outcomes. Although the experimental results indicated that adaptive tutoring did not significantly improve vocabulary test scores (*p* = 0.089), there was a slight improvement in participants' self-reported evaluations of the system's usability (*p* = 0.010) and engagement (*p* = 0.014), suggesting that an adaptive robot tutor may be more adept at maintaining learners' focus. This research offers insights into the design of future robot-assisted language learning systems, particularly in terms of how to personalize teaching strategies by monitoring learners' cognitive states. Van den Berghe et al. (2021) developed an adaptive learning system integrated with a Brain-Computer Interface (BCI) that assesses students' attention levels by monitoring their brainwave activity. The system automatically provides adaptive feedback, such as repeating recently learned words accompanied by symbolic gestures to strengthen memory, when it detects a decrease in students' attention. In the experiment, participants performed learning tasks under two different conditions: one where the robot physically exists and another where the robot appears on the screen in video form. The results showed that under the physical robot condition, participants not only reported higher engagement but also achieved better scores in subsequent vocabulary tests (*t* = 2.86, *p* = 0.004), indicating that the interaction with the physical robot significantly enhanced learning outcomes. Additionally, participants had a more positive impression of the physical robot. The study's conclusion emphasizes the importance of physical interaction in the learning process, especially in adaptive learning environments. It reveals that the physical presence of robots can serve as a powerful teaching tool, improving learning outcomes by increasing student engagement and motivation. The study also highlights the potential application of BCI technology in the design of educational robots, providing a new direction for the design of personalized and interactive learning experiences.

#### 3.2.2 Pronunciation

Krisdityawan et al. (2022) studied the effectiveness of RALL on improving English pronunciation and prosodic skills among Japanese students. Participants were divided into an RALL group using the NAO robot and a traditional e-learning group without RALL, using a computer screen, and underwent three weeks of pronunciation and prosody training. Analysis using independent and paired sample t-tests revealed that although the RALL group showed better performance in English pronunciation (*t* = 0.092, *p* = 0.928, *d* = 0.041), prosody (*t* = 0.092, *p* = 0.109, *d* = −0.351), and overall pronunciation and prosody skills (*t* = −0.271, *p* = 0.790, *d* = −0.121) after learning with the humanoid robot, there were no statistically significant differences between the two groups. Although the study did not find a significant effect of embodiment on RALL effectiveness, the RALL system demonstrated a positive role in improving English pronunciation training for Japanese students, indicating that robot-assisted instruction is an effective language learning tool. Amioka et al. (2023) investigated the potential role of Robot-Assisted Language Learning (RALL) in second language (L2) pronunciation training, particularly for Dutch-speaking learners acquiring Japanese pronunciation. The researchers utilized a social robot to teach 30 Japanese words of varying difficulty and compared three different pronunciation teaching methods: congruent audio-visual speech, incongruent visual speech, and computer-generated audio-visual speech. The experimental results indicated that congruent audio-visual speech did not enhance the learners' pronunciation performance, but instead was inferior to incongruent visual speech [*W* = 16.0, *p* < 0.001, *CLED*(*d*) = 0.25] and computer-generated audio-visual speech [*W* = 76.0, *p* = 0.016, *CLED*(*d*) = 0.38]. This finding challenges the traditional view that audio-visual speech has a positive effect on language learning and pronunciation accuracy. The study suggests that this may be because learners whose native language is not Japanese struggle to effectively lip-read in a non-native environment, thus failing to gain the expected learning benefits from congruent audio-visual speech. Therefore, despite the widely recognized importance of audio-visual speech in language acquisition, its facilitative effect may not be as significant as anticipated in robot-assisted L2 pronunciation learning. Zinina et al. (2023) examined the role of companion robots in Mandarin tone learning, particularly their facilitative effect in pronunciation training. The study found that while auditory cues were more effective than gestural cues in teaching Mandarin tones, the robot's gestural cues enhanced learners' emotional engagement and interest. The experiment involved 20 subjects with no prior knowledge of Mandarin, who learned through two F-2 robots that used both speech and gestures to assist in teaching. The results showed that although auditory cues were more comprehensible when tones were first introduced (*p* < 0.001), all subjects eventually learned the correct tone pronunciation through both types of cues. Furthermore, despite occasional misunderstandings of its gestures, the robot with gestures was considered more comprehensible (*p* < 0.05). The study suggests that social robots can effectively be used to teach Mandarin tones, and future research will focus on developing the best learning strategies that combine speech and gestures to maximize the benefits of robot-assisted language learning.

#### 3.2.3 Speaking and communication

Iio et al. (2019) conducted a study on the effectiveness of RALL systems in improving the English-speaking skills of Japanese adults. Through a seven-day pilot experiment, researchers found that adults using the RALL system showed significant improvements in speaking accuracy, fluency, and pronunciation. The experiment involved nine Japanese female university students who interacted with a robot and used a tablet for 30 min of daily speaking practice. The results indicated that participants became more adept at using English in various contexts, reducing grammatical and lexical errors (*F* = 27.20, *p* < 0.01, *Effect size* = 1.84), increasing speaking speed (*F* = 11.51, *p* < 0.01, *Effect size* = 1.20), shortening silent intervals (*F* = 5.16, *p* < 0.01, *Effect size* = 0.80), and improving pronunciation (*F* = 6.21, *p* < 0.01, *Effect size* = 0.88). This study provides strong evidence that RALL can be an effective tool to help adults enhance their English speaking and communication skills. Later, Iio et al. (2024) explored the comparative efficacy of RALL systems against human tutors in enhancing English speaking skills. The seven-day experimental study involved 26 participants, with 14 interacting with a RALL system and 12 receiving instruction from human tutors. The results of the study revealed that participants in the RALL group exhibited marked improvements in lexical and grammatical accuracy, as well as speech fluency. Statistical analyses indicated a significant reduction in error rates (*F* = 35.15, *p* < 0.001, *Effect size* = 0.302) and an increase in the number of words uttered per second (*F* = 24.12, *p* < 0.001, *Effect size* = 0.176) for the RALL group post-intervention. Additionally, the RALL system's structured approach seemed to enhance participants' pronunciation abilities, although the study did not report specific effect sizes for this parameter. One of the pivotal findings was the RALL system's capacity to diminish speaking anxiety among participants, which is often a barrier to language learning. The structured and non-judgmental nature of the robotic tutor may have contributed to a more relaxed learning environment, thereby promoting better language retention and application. While the RALL system showed promise in improving specific speaking skills, it did not outperform human tutors in all aspects. Notably, there were no significant differences between the two groups in terms of rhythm, complexity, and task achievement, suggesting that human tutors still hold an edge in fostering a nuanced understanding of language and its pragmatic use. Engwall and Lopes (2022) summarized teaching strategies, robot roles, and types from previous RALL studies and proposed a collaborative RALL practice setting for adult learners. Through user research, the authors found that different robot interaction behaviors significantly affected the interaction patterns between the robot and learners, as well as among learners. Specifically, when the robot acted as an “interviewer”, it could guide a clear question-and-answer structure, while the “facilitator” role encouraged interaction among learners, although this required a certain level of language proficiency from the learners. Additionally, the study emphasized the role of peer collaboration in addressing technical and language challenges in communication with robots. Overall, the paper demonstrates that RALL can be an effective tool, enhancing adult learners' speaking practice and communicative skills by providing customized interaction and feedback.

#### 3.2.4 Grammar

In Khalifa et al. (2016), the researchers enhanced learners' grammar training in second language acquisition through simulated multiparty dialogues. The system consisted of two robots, one acting as a teacher and the other as an advanced learner, to simulate the “interactional alignment” phenomenon in human dialogues. This approach encouraged learners to imitate the robots' sample responses, thereby improving the predictability of the Automatic Speech Recognition (ASR) system for the speech of second language (L2) learners. The experiment collected data from the interactions between learners and robots, analyzing how learners could improve their grammatical abilities by mimicking the robots' grammatical structures. The results indicated that this imitation behavior helped learners to naturally acquire grammar without direct instruction, laying the groundwork for the development of advanced language models that can autonomously respond and adapt to learners' responses. Subsequently, in Khalifa et al. (2017), the authors focused on improving second language learners' mastery of specific grammatical structures through repeated inquiry and implicit learning. Similar to their previous work, they developed an “additive” robot-assisted system that utilized two humanoid robots–one as a teacher and the other as an advanced peer learner–to interact with learners. The design included scenarios with repeated questioning aimed at reinforcing learners' understanding and use of three English grammatical expressions (responses to negative questions, passive voice, and causative verbs). The experiment involved 37 Japanese university students. The results showed that while both the experimental and control groups made progress, the experimental group (which was asked questions after the peer robot provided implicit learning answers) showed more significant improvement in correctly using these grammatical expressions. This suggests that the RALL system, by combining tutorial learning and implicit learning, can effectively promote language learners' grammar learning. Continuing their research, Khalifa et al. (2018) used two NAO robots to engage in dialogues with human learners, designing four fixed scenarios that were repeated over four consecutive weeks to help learners implicitly learn specific grammatical patterns, such as causative verbs and inanimate subjects. The experiment was divided into an experimental group and a control group, and the results showed that the experimental group improved in the correct use of grammatical patterns and significantly outperformed their initial state in a retention test conducted weeks after the training. This indicates that the RALL system, through implicit learning, significantly enhanced learners' mastery and long-term memory of grammatical structures, proving that robot assistance not only improves learners' motivation but also effectively promotes grammar learning. Finally, in Khalifa et al. (2019), the researchers designed a Join-in Robot-Assisted Language Learning system (JI-RALL), which involves two robots and one human learner in a three-way dialogue, aiming to improve learners' practical communication skills in real conversational contexts. The study used implicit learning as the main learning method, indirectly teaching language knowledge through dialogues on multiple topics. A series of experiments with 80 participants showed that implicit and repeated learning had a positive effect on improving learners' use of specific grammatical structures. The paper then proposed an improved JI-RALL system method that combines implicit learning and tutoring, enhancing the performance of second language learners at different levels in practical communication skills by adaptively providing corrective feedback. The results suggest that while implicit learning positively affects learners in constructing grammatically appropriate sentences, more repetition and appropriate teaching materials may be needed, especially for beginners, to improve learning outcomes.

### 3.3 Auxiliary effects of affect aspect

The auxiliary effects of affect in RALL have been extensively studied, with a focus on how RALL impacts the emotional aspects of adult learners (Table 3).

**Table 3:** Auxiliary effects of robot-assisted language learning.

**Study**	**A**	**D**	**E**	**M**	**P**	**T**	**U**	**Em**
Kanero et al. (2018)	✓							
de Haas and Conijn (2020)				✓	✓			
Banaeian and Gilanlioglu (2021)	✓		✓		✓	✓		
Alimardani et al. (2022)			✓					
Prinsen et al. (2022)					✓	✓		
Riedmann et al. (2024)			✓	✓	✓			
Vrins et al. (2022)			✓		✓			
Gkinos et al. (2022)					✓	✓		
Zinina et al. (2023)								✓
Engwall and Lopes (2022)					✓			
Lopes et al. (2017)							✓	
Nomoto et al. (2022)			✓					
Khalifa et al. (2019)	✓				✓			
Shen et al. (2019)			✓				

A, attitude toward robots; D, discouragement; E, engagement; M, motivation; P, perception (anthropomorphism, animacy, likability, perceived intelligence, perceived safety); L, Likability, Em, Empathy; T, technology acceptance; U, understandability.

Regarding the emotional impact of robots' feedback on learners, Alimardani et al. (2022) emphasized through experimental data that the motivational gestures of robots in RALL tasks positively affect learners' emotional responses, particularly engagement (E), and indirectly influence learners' attitudes and perceptions toward the robots, which collectively affect the learning outcomes of RALL tasks. de Haas and Conijn (2020) explored how different types of feedback (reward, punishment, and no feedback) affect learners' attitudes (A) and motivation (M) toward robots and how these factors are related to learning outcomes. The study found that punitive feedback was most effective in improving learning outcomes but had lower likability and motivation. Positive feedback (reward) and no feedback had a relatively smaller impact on learning outcomes but had higher likability. Although the type of feedback did not show a significant trend on task motivation, these findings provide insights into the emotional role of feedback mechanisms in robot-assisted learning environments. Banaeian and Gilanlioglu (2021) discussed students' attitudes (A), engagement (E), perceptions of robots (P), and technology acceptance (T) from an experimental data perspective. Through questionnaires and interviews, the study collected students' opinions on the NAO robot, showing that students generally have a positive attitude toward robots, considering them intelligent, safe, and helpful learning tools, indicating a higher level of technology acceptance (T). Interactions with robots increased their engagement, and as a teaching assistant, the robot enhanced students' motivation by providing immediate feedback and repetitive practice. Additionally, students' perceptions of the NAO robot were positive, considering it a friendly and attractive communication partner, involving perceptions of anthropomorphism, perceived intelligence, and perceived safety (P). However, the work did not explicitly mention the affective aspects of frustration (D) and understandability (U). These affective aspects of RALL affect the quality of interaction between students and robots, which may in turn affect language learning outcomes and overall satisfaction with using robot-assisted learning technology. Prinsen et al. (2022) showed that learners under adaptive tutoring conditions reported increased system usability and engagement, indicating that adaptive teaching might be more effective in maintaining learners' focus and interest. The study also used the Godspeed questionnaire to assess learners' perceptions and attitudes toward robots (P), including anthropomorphism, liveliness, cuteness, perceived intelligence, and perceived safety. The results showed a slight increase in some dimensions of learners' perceptions of robots (such as liveliness and cuteness) under adaptive tutoring conditions, although these increases were not statistically significant. Additionally, the study indirectly measured learners' technology acceptance (T) through the System Usability Scale (SUS) questionnaire, which focuses on the technical usability of the system, that is, learners' perception of the ease of using the system. Kanero et al. (2018) found that participants' negative attitudes (A) toward robots, especially concerns about the potential negative societal impact of robots (NARS's S2 subscale), were negatively correlated with scores in all four tests (immediate and delayed productive and receptive tests). Riedmann et al. (2024) discussed the complex and not always positive role of the affective aspects of engagement (E), motivation (M), and perceptions of robots (P) in RALL. The integration of social robots did not increase engagement and motivation as expected, and learners' perceptions of the robots' intelligence were positively correlated with their learning experience, indicating that the interactive design of robots and learners' emotional reactions need to be considered when designing RALL tasks. Vrins et al. (2022) directly explored the emotional aspects in RALL tasks, including perceptions of robots (P) and learners' engagement (E). Using the Godspeed Robot Impression Questionnaire and the User Engagement Scale, the study found that interactions with a physical robot significantly improved students' positive attitudes toward robots, increased engagement and motivation, and enhanced perceptions such as anthropomorphism, liveliness, friendliness, perceived intelligence, and perceived safety. These improvements in emotional aspects positively affected RALL, potentially increasing students' acceptance of using robot-assisted learning technology and improving learning outcomes. Zinina et al. (2023) mentioned that robots with gesture cues were considered more likable (P) and elicited more empathy (Em), indicating the emotional impact of perceptions of robots. Khalifa et al. (2019) showed that using the RALL system improved learners' attitudes (A) toward robots. In terms of perceptions of robots (P), learners considered the robots to have good interaction capabilities and to understand their interaction intentions. Lopes et al. (2017) believed that in terms of understandability (U), although there are challenges for robots to understand learners' language, the responses of the robots need to be clear and understandable. This affective aspect of RALL can affect the quality of interaction between learners and robots, thereby affecting language learning outcomes and the overall learning experience. Engwall and Lopes (2022) demonstrated that when robots played more personified roles in the dialogue, learners' perceptions (P) of the robots, such as anthropomorphism, approachability, and perceived intelligence, affected the quality of their interaction and learning experience. Gkinos et al. (2022) showed that participants generally had a positive attitude toward Socially Assistive Robots (SAR), appreciating SAR as an interesting, innovative, and interactive tool, indicating that a positive attitude toward robots is crucial for the success of RALL. Additionally, AR was designed as a culturally characterized role, which increased learners' acceptance (P) and perceived intelligence and safety (T), beneficial for the acceptance and effectiveness of RALL. Nomoto et al. (2022) found that a physical robot language learning solution seems promising in terms of increased engagement (E) levels, but technical difficulties greatly reduce the practicality of using robots to learn. Shen et al. (2019) showed that after using the Embodied Teaching Assistant Robot (ETAR), students' motivation questionnaires indicated a more positive attitude toward robots, a greater willingness to interact with them, and feeling more comfortable and confident in the company of the robot. Students' engagement increased, as evidenced by more frequent oral practice and classroom interaction with ETAR. In terms of motivation, students showed a significant increase in interest and drive to learn English after interacting with ETAR, being more willing to invest time and effort to improve their speaking skills. Perceptions of the robot also became more positive, with students finding ETAR fun, intelligent, and safe and reliable, promoting their acceptance and enjoyment of this new type of teaching tool. These positive changes in affective aspects indicate that RALL technology plays an important role in improving students' language learning experience and outcomes.

### 3.4 Effects of instruction type

The effects of social interaction type in Robot-Assisted Language Learning (RALL) are significant, as the way in which robots express instructional cues can greatly influence the assistance provided to foreign language learning. The main types of expression are explicit and implicit.

#### 3.4.1 Explicit instruction

Prinsen et al. (2022) utilized robots that adjust their teaching behavior based on real-time monitoring of learners' EEG signals, demonstrating explicit instruction. Although this approach helps maintain attention and improves perceived system usability, it did not significantly enhance vocabulary test scores. Banaeian and Gilanlioglu (2021) experimented with students directly asking the robot for the meaning of target vocabulary, with the robot providing definitions, examples, comprehension checks, and feedback. This explicit instruction facilitated meaningful exchanges and improved vocabulary learning and memory. However, some students reported issues with speech recognition and speech rate, indicating the importance of technological refinement for interaction efficiency and learning outcomes. Alimardani et al. (2022) provided explicit verbal feedback and motivational gestures to learners. While this increased self-reported engagement, it did not significantly improve vocabulary test performance or cognitive engagement as measured by the EEG Engagement Index, suggesting a need to consider the relationship between interaction type and learning outcomes. Kanero et al. (2018) used structured steps to explicitly teach target words, requiring active participation. Negative attitudes toward robots, particularly concerns about societal impact, were linked to poorer learning outcomes in this explicit instruction environment. Van den Berghe et al. (2021) designed robots to adaptively respond to decreased student attention with explicit instructions like repeating words and symbolic gestures. Robots combined with this explicit instruction significantly enhanced student engagement and vocabulary learning outcomes compared to video robots on a screen. Riedmann et al. (2024) had robots guide learners explicitly through learning applications, but this did not significantly improve engagement or intrinsic motivation, contrary to hypotheses. Amioka et al. (2023) required learners to mimic and repeat Japanese words demonstrated by robots. Explicit instruction did not improve pronunciation accuracy as expected, even with consistent audio-visual feedback. Krisdityawan et al. (2022) used the robot NAO as a teacher, providing explicit instructions and demonstrations for English pronunciation and prosody to Japanese high school students. This explicit instruction positively impacted pronunciation and prosody skills, especially in articulation. Zinina et al. (2023) taught Mandarin tones with explicit gestures visualizing pitch patterns, which increased engagement despite the initial effectiveness of verbal cues. Gkinos et al. (2022) found that robots as interactive partners with immediate feedback and encouragement simulated real conversations, enhancing interest and attention in language learning. Nomoto et al. (2022) created an interactive application system with robots as explicit language learning tools through pre-programmed dialogues. Shen et al. (2019) used explicit teaching tasks like roll-calling, pronunciation practice, and reading exercises, which increased learning motivation in various areas.

#### 3.4.2 Implicit instruction

Implicit instruction, as introduced in Section 1.4, favors a bottom-up approach to language learning. It differs from explicit instruction by not providing direct teaching of grammatical rules. Instead, it encourages learners to discover language through exposure and interaction, promoting an intuitive understanding of the language. For example, rather than telling a learner the grammar for ordering coffee, a robot might simulate a café scenario where the learner must order coffee through role-play. This immersive approach allows adults to naturally absorb language patterns, aligning with their preference for self-discovery and internalization of linguistic structures.

Khalifa et al. (2016, 2017, 2019) and Kanero et al. (2018) proposed a join-in RALL model with one robot as a teacher and another as an advanced learner. This model significantly enhanced learners' performance in implicit learning environments and maintained high learning outcomes in retention tests. The study emphasized the need for dynamic dialogue difficulty adjustment and adaptive discourse control to improve learning efficiency and motivation. Engwall and Lopes (2022) designed robots as “interviewers” and “facilitators,” increasing interactivity through turn-taking questions and encouraging learner dialogue. Implicit instruction designs effectively enhanced language practice opportunities and engagement. Lopes et al. (2017) analyzed the robot as a language practice partner from an implicit instruction perspective. Structured and slightly repetitive interaction patterns provided by the robot as a learning partner were considered beneficial for beginner language learners (A1 to A2 levels). Iio et al. (2019, 2024) interacted with robots through tablet instructions, but the robot only played the role of the user's dialogue partner without providing any learning suggestions, encouraging learners to engage in conversation and learn through interaction, enhancing spoken English skills.

In summary, both explicit and implicit instruction types in RALL have their merits and challenges. Explicit instructions provide clear instructions and structured feedback, which can enhance engagement but may require technological refinement for better learning outcomes. Implicit instructions, on the other hand, offer a more naturalistic learning environment that can improve language acquisition and long-term retention, especially when combined with peer collaboration and adaptive teaching strategies.

## 4 Discussion

### 4.1 Response to RQ1: latest advancements of RALL for adult second language learning

#### 4.1.1 Improvement in oral and communicative competence

RALL has shown significant effects on improving the oral and communicative abilities of adults. Studies by Engwall and Lopes (2022), Gkinos et al. (2022), Nomoto et al. (2022), Iio et al. (2019), Shen et al. (2019) indicate that interacting with robots allows learners to practice speaking in a low-stress environment, enhancing fluency and confidence. Immediate feedback and structured dialogue exercises, as mentioned by Lopes et al. (2017), provide opportunities for repetitive practice and instant correction, which are crucial for improving speaking skills.

A key advantage of RALL is its capacity to offer immediate and interactive learning experiences. Robots can respond in real-time to learners' language output and communicate through various interaction modes (e.g., voice, text, gestures). This interactivity not only promotes language practice but also increases engagement and motivation, as Alimardani et al. (2022) pointed out, with motivational gestures and verbal feedback from robots enhancing learner engagement.

Despite the positive outcomes, technical limitations remain a significant challenge. The accuracy of speech recognition and synthesis directly affects effective communication between robots and learners. As Amioka et al. (2023) noted, inaccurate speech recognition or synthesis can hinder learners' understanding of robot feedback, affecting learning outcomes. Additionally, response time is critical; delays can disrupt fluid conversation and reduce the naturalness of interaction. Therefore, optimizing related technologies is essential for the development of RALL. Advances in AI and machine learning have significantly improved robots' speech recognition and synthesis capabilities, offering more natural and effective interaction experiences. Adaptive learning algorithms, as explored by Prinsen et al. (2022), can adjust teaching content and difficulty in real time based on learner performance, providing a more personalized learning experience.

#### 4.1.2 Inconsistency in vocabulary, pronunciation, and grammar learning outcomes

The effectiveness of RALL in vocabulary, pronunciation, and grammar learning shows some inconsistency. Some studies, such as Vrins et al. (2022), suggest that physical robots are more effective in enhancing vocabulary learning outcomes than video robots. However, other studies, including Prinsen et al. (2022) and Banaeian and Gilanlioglu (2021), did not find significant improvements in learning outcomes. This inconsistency may stem from various factors, including robot design, teaching methods, learner characteristics, and differences in experimental design.

To improve the consistency and effectiveness of RALL in vocabulary, pronunciation, and grammar learning, it is necessary to integrate educational theory with technology. Educational theory can guide the teaching strategies and interaction design of robots, ensuring they align with language learning principles. Technological integration should consider learners' needs and backgrounds, as well as specific teaching environment requirements. For example, Khalifa et al.'s research indicates that through implicit learning and communication with robots, learners can naturally acquire grammar without direct instruction.

In summary, RALL shows potential in enhancing oral and communicative abilities in adult language learning, despite facing challenges from technical limitations. Technological advancements and the integration of educational theory are crucial for improving RALL effectiveness. Future research needs to further explore how to optimize robot design and teaching strategies and overcome technical limitations to provide more effective and consistent language learning experiences for adults.

### 4.2 Response to RQ2: implicit vs. explicit effectiveness

#### 4.2.1 Impact of robot roles on learning outcomes

When examining the effectiveness of instructional vs. companionship robots in RALL, it is evident that the role of the robot significantly affects adult language learning outcomes. Instructional robots often take on the role of a teacher, providing structured teaching and clear guidance, while companionship robots act as peers to learners, promoting language acquisition through implicit learning and interaction. According to research by Khalifa et al., companionship robots can more effectively facilitate grammar learning by simulating real communication scenarios and providing implicit feedback. This learning approach aligns with socio-constructivist theory, which posits that knowledge is constructed through social interaction and communication. In RALL, companionship robots offer an interactive platform where learners can naturally learn and use language in communication with the robot. This interaction includes not only the transfer of language knowledge but also the exchange of emotions, attitudes, and cultural aspects. As shown by Engwall and Lopes (2022), robots designed as “interviewers” and “facilitators” increase interactivity by taking turns in questioning and encouraging dialogue among learners, thereby enhancing language practice opportunities and engagement.

#### 4.2.2 Importance of implicit instruction

Implicit interaction plays a significant role in RALL. Unlike explicit learning, which relies on direct instruction and clear feedback, implicit interaction is achieved through observation, imitation, and practice. In RALL, learners absorb language knowledge implicitly through natural interaction with robots. As noted in Khalifa et al.'s research, learners can naturally learn grammar without direct instruction by mimicking sample responses provided by robots. This learning method is more in line with adult learning habits as it allows for autonomous exploration and learning in real communication scenarios. Moreover, to facilitate language acquisition, the robot's interactive behavior needs to be carefully designed. Firstly, robots must provide timely and appropriate feedback to enhance language practice. Secondly, the design of implicit instruction should encourage learner participation and exploration. Additionally, anthropomorphic features of robots, such as friendliness, perceived intelligence, and safety, also affect the quality of interaction and learning experience. As demonstrated in Shen et al. (2019), students showed a more positive attitude, increased engagement, and enhanced motivation after interacting with anthropomorphized robots.

In conclusion, companionship robots have shown higher effectiveness in adult language learning, consistent with socioconstructivist theory and the importance of implicit learning. Future RALL research and practice should focus on designing interactive behaviors of robots to promote language acquisition and improve learner engagement and performance. It is also necessary to further explore how different types of robot roles can adapt to the needs and backgrounds of different learners and integrate educational theory and technology in RALL to achieve the best teaching outcomes.

### 4.3 Response to RQ3: robot traits that promote RALL performance

#### 4.3.1 Feedback mechanisms and anthropomorphic features

In RALL, feedback mechanisms are key to enhancing learner engagement and motivation. Studies by Alimardani et al. (2022), Vrins et al. (2022), Zinina et al. (2023), Gkinos et al. (2022) indicate that robots capable of providing timely feedback or encouragement significantly improve learner engagement and motivation. This feedback includes not only verbal responses but also gestures and vocal expressions, which enrich the interaction between learner and robot, making the learning process more dynamic and enjoyable.

Anthropomorphic features are another critical aspect of robot design. The appearance, behavior, and communication style of robots can influence how learners perceive and accept them. de Haas and Conijn (2020) found that robots with higher anthropomorphic traits can provide more effective learning feedback, although this might affect some emotional responses. Anthropomorphism makes robots not only technological tools but also companions that can evoke emotional responses from learners.

#### 4.3.2 Emotional computing and personalized interaction

The application of emotional computing and personalized interaction in robot design is essential for enhancing RALL performance. Robots need to recognize and respond to learners' emotional states to offer more personalized and effective learning experiences. Prinsen et al. (2022) demonstrated the importance of emotional computing in RALL by adjusting teaching behaviors based on real-time monitoring of learners' EEG signals. Moreover, personalized feedback, including verbal, gestural, and vocal responses, is another core feature of RALL that can improve learner engagement, motivation, and attention (Alimardani et al., 2022; Vrins et al., 2022; Zinina et al., 2023; Gkinos et al., 2022).

To achieve personalized feedback, factors such as learners' cultural backgrounds and learning styles must be considered. Kanero et al. (2018) noted that individuals with negative attitudes toward robots may perform poorly in explicit interactive learning environments, indicating that personalized learning strategies need to account for learners' emotional responses and attitudes. Through personalized learning, robots can offer more flexible and dynamic approaches to cater to the needs of different learners.

In summary, feedback mechanisms, anthropomorphic features, and personalized feedback are crucial traits that promote RALL performance. Future RALL research and practice should focus on optimizing these traits to provide more effective and engaging language learning experiences.

### 4.4 Future research questions (RQs) for adult RALL

#### 4.4.1 Current limitations

While there has been progress in the application of RALL for adults, there are still limitations.

Most current RALL methods rely on explicit instruction, which often requires learners to depend on direct guidance and feedback from robots. For example, Banaeian and Gilanlioglu (2021) found that although students could obtain definitions and examples of vocabulary by asking the robot, this direct dependence might limit learners' ability to explore and affect their initiative. Additionally, explicit instruction may not promote deep learning. Prinsen et al. (2022) showed that adaptive tutoring, while maintaining learners' attention, did not significantly improve vocabulary test scores, suggesting that explicit instruction may not stimulate deeper cognitive processing. Moreover, the effectiveness of explicit instruction is too limited by technical capabilities. Technical issues such as speech recognition problems and fast speech rates (as described by Banaeian and Gilanlioglu, 2021) may affect learners' understanding of the robot and the quality of interaction. Most importantly, explicit instruction may not be sufficient to maintain long-term motivation and engagement. Alimardani et al. (2022) pointed out that although motivational gestures increased self-reported engagement, they did not translate into improved vocabulary test performance, indicating that explicit instruction may require more strategies and approaches to stimulate intrinsic motivation.

Implicit instruction can provide a more natural communication environment and encourage autonomous learning. Khalifa et al. showed that through simulated multiparty dialogues and implicit learning, learners could naturally learn grammar without direct guidance. Implicit interaction also promotes social interaction among learners. Lopes et al. (2017) found that robots as language practice partners could provide structured and slightly repetitive interaction patterns, which are helpful for the language practice of beginners. In terms of personalized needs, implicit instruction can also adapt well. Kanero et al. (2018) showed that despite users having a negative attitude toward the social impact of robots, the design of implicit instruction could adapt to different learners' attitudes and motivations. Moreover, implicit instruction helps to enhance learners' long-term memory of language knowledge. Khalifa et al. (2018) showed that through implicit learning, learners improved in the correct use of grammatical patterns and showed significant improvement in retention tests weeks after training.

However, although implicit instruction seems to have many advantages, there are still some issues to be resolved.

- **Neglect of learner emotions:** implicit instruction, while providing a more natural communication environment, may neglect the emotional needs of learners and cannot provide direct feedback on emotional issues.
- **Lack of social interaction:** implicit instruction may not fully utilize the robot to promote social interaction among learners. In the study by Lopes et al. (2017), the robot as a language practice partner lacked the ability to promote interaction among learners.
- **Insufficient cross-cultural adaptability:** implicit instruction may have limitations in cross-cultural adaptability, as cultural differences have varying degrees of understanding of implicit instruction. Shen et al. (2019) also pointed out that although the ETAR robot improved learning motivation, support for cross-cultural communication and adaptability may be insufficient.

#### 4.4.2 Potential research questions

Despite the progress made, the application of RALL for adult learners still has some limitations, mainly manifested in the over-reliance on explicit instruction, limitations of technical capabilities, insufficient stimulation of learners' intrinsic motivation, neglect of emotional participation, lack of social interaction, insufficient cross-cultural adaptability, and limited impact on long-term memory and learning transfer. To overcome these limitations and further improve the teaching effectiveness of RALL systems, future research can focus on the following research questions:

##### 4.4.2.1 PRQ1: how to integrate explicit and implicit instructions to enhance learning outcomes?

The integration of explicit and implicit instructions is key to improving the effectiveness of RALL. Explicit instruction provides clear guidance and immediate feedback (Kanero et al., 2018; de Haas and Conijn, 2020; Banaeian and Gilanlioglu, 2021), which is suitable for structured learning environments and beginners. Implicit instruction, by simulating real communication scenarios, encourages learners to explore and learn autonomously (Lopes et al., 2017; Engwall and Lopes, 2022; Khalifa et al., 2016, 2017, 2018, 2019). Future research can explore the best combination of these two interaction modes at different learning stages, such as using more explicit instruction in the beginner stage and gradually increasing the proportion of implicit instruction as learners' abilities improve. Additionally, research can customize personalized interaction strategies based on learners' personalities, abilities, and styles to achieve personalized learning.

##### 4.4.2.2 PRQ2: how to design RALL systems to better stimulate learners' intrinsic motivation?

Intrinsic motivation is a key factor in driving learners to continue learning and progress (Ho and Lim, 2020; Bakhtiar and Hadwin, 2022). The design of RALL systems should consider how to stimulate learners' intrinsic motivation. Research can explore factors affecting learners' intrinsic motivation, such as personal interests, self-efficacy, and the supportiveness of the learning environment. Gamification learning elements, such as challenges, rewards, and feedback, can be integrated with RALL systems to enhance learner engagement and motivation. Moreover, innovative feedback mechanisms, such as peer evaluation, self-reflection, and metacognitive strategies, can provide more constructive and motivational feedback.

##### 4.4.2.3 PRQ3: how can implicit instruction promote learners' emotional engagement?

Emotional engagement plays a vital role in the learning process (Nomoto et al., 2022). As we introduced previously, implicit instruction helps with learners' emotional engagement by providing a more natural communication environment. To realize it, affective computing technology can be applied to RALL systems to identify and respond to learners' emotional states, providing more personalized and empathetic interactions. Research can analyze emotional design elements in implicit instruction, such as storytelling, situation simulation, and role-playing, enhancing learners' emotional experience and engagement. Additionally, exploring how to improve learners' emotional intelligence and social skills through implicit instruction is an important direction for future research.

##### 4.4.2.4 PRQ4: how to strengthen social interaction among learners through RALL systems?

Social interaction is an essential component of language learning (Jarvis, 2011; Dornyei, 2013; Kanero et al., 2018; Lopes et al., 2017). RALL system design should consider how to promote collaborative learning and social interaction among learners. Research can explore collaborative learning theories and design interactive activities that promote learner communication and cooperation. Robots as mediators can facilitate communication among learners, providing feedback and support. Moreover, researching the differences in learner interaction across different sociocultural backgrounds and optimizing RALL systems to adapt to these differences is crucial for enhancing the quality of social interaction.

##### 4.4.2.5 PRQ5: how to improve the cross-cultural adaptability of RALL systems?

Cross-cultural adaptability is an important consideration in a global language learning environment (Hofstede, 1986). Research needs to focus on how cultural differences affect learners' interaction with RALL systems and how to design RALL systems that can adapt to different cultural backgrounds. The cultivation of intercultural communication skills can be integrated into RALL system design, providing multicultural content and communication opportunities to enhance learners' intercultural awareness and capabilities. Additionally, localization and cultural adaptability design, such as adapting to language, customs, and social norms, can make RALL systems more attuned to the needs of learners from different cultural backgrounds.

## 5 Conclusion

The rapid development of artificial intelligence technology has introduced Robot-Assisted Language Learning (RALL) as an innovative educational model, offering new perspectives and possibilities for adult language learning. This summary reviews the current state of RALL applications in adult language learning, the impact of different types of interactions on learning outcomes, how robot traits can enhance RALL performance, and suggests directions for future research.

### 5.1 Current status of RALL applications

RALL has shown significant effectiveness in improving the oral and communicative abilities of adults. Through interaction with robots, learners can practice speaking in a low-stress environment, enhancing linguistic fluency and confidence. The immediate feedback and structured dialogue exercises provided by robots offer opportunities for repetitive practice and instant correction, which are crucial for improving speaking skills. However, the effectiveness of RALL in vocabulary, pronunciation, and grammar learning has shown some inconsistency, possibly due to differences in robot design, teaching methods, learner characteristics, and experimental design.

#### 5.1.1 Impact of interaction types

Both instructional and companionship robots play a role in RALL, but companionship robots can more effectively facilitate grammar learning by simulating real communication scenarios and providing implicit feedback, aligning with socio-constructivist theory. Implicit instruction, which does not rely on direct guidance and explicit feedback, is achieved through observation, imitation, and practice, fitting the learning habits of adults. However, explicit instruction may limit learners' ability to explore independently and affect their initiative, and may not promote deep learning.

#### 5.1.2 Robot affective traits

The feedback mechanisms, anthropomorphic features, and personalized feedback of robots are important traits that promote RALL performance. Timely feedback can improve learner engagement and motivation, anthropomorphic features can affect how learners perceive and accept robots, and personalized feedback can adapt to the needs of different learners. The application of affective computing and personalized interaction is crucial for enhancing RALL performance, as robots need to recognize and respond to learners' emotional states to provide a more personalized and effective learning experience.

### 5.2 Future research directions

In light of the current limitations of RALL systems, future research can delve deeper into the following areas:

- **Integration of explicit and implicit instructions:** explore the optimal combination of these interaction modes at different learning stages and customize personalized interaction strategies based on learners' personalities, abilities, and styles.
- **Stimulating intrinsic motivation:** investigate factors affecting intrinsic motivation, integrate gamification elements, and innovate feedback mechanisms.
- **Promoting emotional engagement:** apply affective computing technology, analyze emotional design elements, and explore how implicit instruction can enhance learners' emotional intelligence and social skills.
- **Strengthening social interaction:** explore collaborative learning theories, design interactive activities that promote learner communication and cooperation, and optimize RALL systems to adapt to differences in learner interaction across various sociocultural backgrounds.
- **Improving cross-cultural adaptability:** focus on how cultural differences affect interaction and design RALL systems that can adapt to different cultural backgrounds, fostering intercultural communication skills.

As an innovative language learning tool, RALL offers more flexible and personalized learning methods for adults. Despite technical challenges and considerations for learners' emotional needs, RALL has the potential to become an important auxiliary means in the field of language learning with technological advancements and deeper integration of educational theory. Psychologically, RALL's interactive and structured pedagogical model is well-positioned to ameliorate cognitive load, in accordance with cognitive load theory. Moreover, it is adept at nurturing intrinsic motivation–a pivotal component for the persistence and success of language learning endeavors. Although RALL confronts technical challenges and must account for the affective dimensions of the learning experience, its potential to serve as an indispensable adjunct in language education is evident. To harness the full potential of RALL, future scholarly inquiry and pedagogical practice ought to concentrate on the refinement of interactive modalities, augmentation of technological prowess, invigoration of intrinsic motivation, enhancement of emotional engagement, fortification of social interactivity, and optimization of cross-cultural adaptability. These concerted efforts are essential for surmounting existing limitations and attaining superior pedagogical outcomes through RALL systems. By doing so, RALL can be leveraged to deliver efficient and captivating language learning experiences for adults, adeptly meeting the dynamic and expanding demands of the global linguistic learning landscape.

## References

[B1] Aidinlou N. A. Alemi M. Farjami F. Makhdoumi M. (2014). Applications of robot assisted language learning (rall) in language learning and teaching. Teach. Learn. 2, 12–20. 10.11648/j.ijll.s.2014020301.12

[B2] Alemi M. Meghdari A. Ghazisaedy M. (2014). Employing humanoid robots for teaching english language in iranian junior high-schools. Int. J. Humanoid Robot. 11:1450022. 10.1142/S0219843614500224

[B3] Alemi M. Meghdari A. Ghazisaedy M. (2015). The impact of social robotics on L2 learners' anxiety and attitude in english vocabulary acquisition. Int. J. Social Robot. 7, 523–535. 10.1007/s12369-015-0286-y

[B4] Alimardani M. Harinandansingh J. Ravin L. de Haas M. (2022). “Motivational gestures in robot-assisted language learning: a study of cognitive engagement using EEG brain activity,” in 2022 31st IEEE International Conference on Robot and Human Interactive Communication (RO-MAN) (Napoli: IEEE), 1393–1398.

[B5] Amioka S. Janssens R. Wolfert P. Ren Q. Pinto Bernal M. J. Belpaeme T. (2023). “Limitations of audiovisual speech on robots for second language pronunciation learning,” in Proceedings of the 2023 ACM/IEEE International Conference on Human-Robot Interaction (Stockholm: IEEE), 359–367.

[B6] Bakhtiar A. Hadwin A. F. (2022). Motivation from a self-regulated learning perspective: application to school psychology. Can. J. School Psychol. 37, 93–116. 10.1177/0829573521105469928648240

[B7] Banaeian H. Gilanlioglu I. (2021). Influence of the nao robot as a teaching assistant on university students' vocabulary learning and attitudes. Aust. J. Educ. Technol. 37, 71–87. 10.14742/ajet.6130

[B8] Belpaeme T. Vogt P. Van den Berghe R. Bergmann K. Göksun T. De Haas M.. (2018). Guidelines for designing social robots as second language tutors. Int. J. Soc. Robot. 10, 325–341. 10.1007/s12369-018-0467-6 30996752 PMC6438435

[B9] de Haas M. Conijn R. (2020). “Carrot or stick: the effect of reward and punishment in robot assisted language learning,” in HRI '20: ACM/IEEE International Conference on Human-Robot Interaction (Cambridge), 177–179.

[B10] Dornyei Z. (2013). The Psychology of Second Language Acquisition. Oxford: Oxford University Press.

[B11] Engwall O. Lopes J. (2022). Interaction and collaboration in robot-assisted language learning for adults. Comp. Assist. Lang. Learn. 35, 1273–1309. 10.1080/09588221.2020.1799821

[B12] Engwall O. Lopes J. Åhlund A. (2021). Robot interaction styles for conversation practice in second language learning. Int. J. Soc. Robot. 13, 251–276. 10.1007/s12369-020-00635-y

[B13] Gkinos M. Velentza A.-M. Fachantidis N. (2022). “Utilization of socially assistive robot's activity for teaching pontic dialect,” in International Conference on Human-Computer Interaction (Cham: Springer), 486–505. 10.1007/978-3-031-05409-9_36

[B14] Gordon G. Spaulding S. Westlund J. K. Lee J. J. Plummer L. Martinez M.. (2016). “Affective personalization of a social robot tutor for children's second language skills,” in Proceedings of the AAAI Conference on Artificial Intelligence (Washington, DC: AAAI Press).

[B15] Han J.-H. Jo M.-H. Jones V. Jo J.-H. (2008). Comparative study on the educational use of home robots for children. J. Inform. Proc. Syst. 4, 159–168. 10.3745/JIPS.2008.4.4.159

[B16] Ho Y. Y. Lim W. Y. R. (2020). “Educating adult learners: bridging learners' characteristics and the learning sciences,” in Diversity and Inclusion in Global Higher Education: Lessons from Across Asia, 97–115.

[B17] Hofstede G. (1986). Cultural differences in teaching and learning. Int. J. Intercultur. Relat. 10, 301–320. 10.1016/0147-1767(86)90015-5

[B18] Iio T. Maeda R. Ogawa K. Yoshikawa Y. Ishiguro H. Suzuki K.. (2019). Improvement of japanese adults' english speaking skills via experiences speaking to a robot. J. Comp. Assist. Learn. 35, 228–245. 10.1111/jcal.12325

[B19] Iio T. Yoshikawa Y. Ogawa K. Ishiguro H. (2024). Comparison of outcomes between robot-assisted language learning system and human tutors: focusing on speaking ability. Int. J. Soc. Robot. 16, 743–761. 10.1007/s12369-024-01134-0

[B20] Jarvis P. (2011). “Adult learning: andragogy versus pedagogy or from pedagogy to andragogy,” in The Routledge International Handbook of Learning (London: Routledge), 154–163.

[B21] Kanda T. Hirano T. Eaton D. Ishiguro H. (2004). Interactive robots as social partners and peer tutors for children: a field trial. Human-Comp. Interact. 19, 61–84. 10.1207/s15327051hci1901&amp;2_4

[B22] Kanero J. Franko I. Oranç C. Uluşahin O. Koşkulu S. Adıgüzel Z.. (2018). “Who can benefit from robots? effects of individual differences in robot-assisted language learning,” in 2018 Joint IEEE 8th International Conference on Development and Learning and Epigenetic Robotics (ICDL-EpiRob) (Tokyo: IEEE), 212–217.

[B23] Kennedy J. Baxter P. Senft E. Belpaeme T. (2016). “Social robot tutoring for child second language learning,” in 2016 11th ACM/IEEE International Conference on Human-Robot Interaction (HRI) (Christchurch: IEEE), 231–238.

[B24] Khalifa A. Kato T. Yamamoto S. (2016). “Joining-in-type humanoid robot assisted language learning system,” in Proceedings of the Tenth International Conference on Language Resources and Evaluation (LREC'16) [Portorož: European Language Resources Association (ELRA)], 245–249.

[B25] Khalifa A. Kato T. Yamamoto S. (2017). Measuring effect of repetitive queries and implicit learning with joining-in-type robot assisted language learning system. SLaTE 2017, 13–17. 10.21437/SLaTE.2017-3

[B26] Khalifa A. Kato T. Yamamoto S. (2018). “The retention effect of learning grammatical patterns implicitly using joining-in-type robot-assisted language-learning system,” in Text, Speech, and Dialogue: 21st International Conference, TSD 2018 (Brno: Springer), 492–499.

[B27] Khalifa A. Kato T. Yamamoto S. (2019). Learning effect of implicit learning in joining-in-type robot-assisted language learning system. Int. J. Emerg. Technol. Learn. 14:9212. 10.3991/ijet.v14i02.9212

[B28] Krisdityawan E. Yokota S. Matsumoto A. Chugo D. Muramatsu S. Hashimoto H. (2022). “Effect of embodiment and improving japanese students' english pronunciation and prosody with humanoid robot,” in 2022 15th International Conference on Human System Interaction (HSI) (Melbourne: IEEE), 1–6.

[B29] Lee H. Lee J. H. (2022). The effects of robot-assisted language learning: a meta-analysis. Educ. Res. Rev. 35:100425. 10.1016/j.edurev.2021.100425

[B30] Lee S. Noh H. Lee J. Lee K. Lee G. G. Sagong S.. (2011). On the effectiveness of robot-assisted language learning. ReCALL 23, 25–58. 10.1017/S0958344010000273

[B31] Lopes J. Engwall O. Skantze G. (2017). “A first visit to the robot language café,” in ISCA Workshop on Speech and Language Technology in Education [International Speech Communication Association (ISCA Archive)].

[B32] Mazzoni E. Benvenuti M. (2015). A robot-partner for preschool children learning english using socio-cognitive conflict. J. Educ. Technol. Soc. 18, 474–485.

[B33] Mubin O. Shahid S. Bartneck C. (2013). Robot assisted language learning through games: a comparison of two case studies. Austr. J. Intellig. Inform. Process. Syst. 13, 9–14.

[B34] Nomoto M. Lustig A. Cossovich R. Hargis J. (2022). “Qilin, a robot-assisted chinese language learning bilingual chatbot,” in Proceedings of the 4th International Conference on Modern Educational Technology (New York, NY: Association for Computing Machinery), 13–19.

[B35] Park S. J. Han J. H. Kang B. H. Shin K. C. (2011). “Teaching assistant robot, robosem, in english class and practical issues for its diffusion,” in Advanced Robotics and its Social Impacts (Menlo Park, CA: IEEE), 8–11.

[B36] Prinsen J. Pruss E. Vrins A. Ceccato C. Alimardani M. (2022). “A passive brain-computer interface for monitoring engagement during robot-assisted language learning,” in 2022 IEEE International Conference on Systems, Man, and Cybernetics (SMC) (Menlo Park, CA: IEEE), 1967–1972.

[B37] Randall N. (2019). A survey of robot-assisted language learning (rall). ACM Trans. Human-Robot Interact. 9, 1–36. 10.1145/3345506

[B38] Riedmann A. Schaper P. Lugrin B. (2024). Integration of a social robot and gamification in adult learning and effects on motivation, engagement and performance. AI & *Soc*. 39, 369–388. 10.1007/s00146-022-01514-y

[B39] Schodde T. Bergmann K. Kopp S. (2017). “Adaptive robot language tutoring based on bayesian knowledge tracing and predictive decision-making,” in Proceedings of the 2017 ACM/IEEE International Conference on Human-Robot Interaction (Vienna: IEEE), 128–136.

[B40] Shen W.-W. Tsai M.-H. M. Wei G.-C. Lin C.-Y. Lin J.-M. (2019). “ETAR: an english teaching assistant robot and its effects on college freshmen's in-class learning motivation,” in International Conference on Innovative Technologies and Learning (Cham: Springer), 77–86.

[B41] Tanaka F. Matsuzoe S. (2012). Children teach a care-receiving robot to promote their learning: field experiments in a classroom for vocabulary learning. J. Human-Robot Interact. 1, 78–95. 10.5898/JHRI.1.1.Tanaka28396741

[B42] Van den Berghe R. Oudgenoeg-Paz O. Verhagen J. Brouwer S. De Haas M. De Wit J.. (2021). Individual differences in children's (language) learning skills moderate effects of robot-assisted second language learning. Front. Robot. AI 8:676248. 10.3389/frobt.2021.676248 34504871 PMC8421643

[B43] Van den Berghe R. Verhagen J. Oudgenoeg-Paz O. Van der Ven S. Leseman P. (2019). Social robots for language learning: a review. Rev. Educ. Res. 89:259–295. 10.3102/003465431882128638293548

[B44] Vrins A. Pruss E. Prinsen J. Ceccato C. Alimardani M. (2022). “Are you paying attention? The effect of embodied interaction with an adaptive robot tutor on user engagement and learning performance,” in International Conference on Social Robotics (Cham: Springer), 135–145.

[B45] Zhang Z. (2022). Learner engagement and language learning: a narrative inquiry of a successful language learner. Lang. Learn. J. 50, 378–392. 10.1080/09571736.2020.1786712

[B46] Zinina A. Kotov A. Arinkin N. Gureyeva A. (2023). “Can a robot companion help students learn chinese tones? the role of speech and gesture cues,” in Conference on Creativity in Intelligent Technologies and Data Science (Cham: Springer), 420–432.

